# An Apparent Lack of Epidemiologic Association between Hepatitis C Virus Knowledge and the Prevalence of Hepatitis C Infection in a National Survey in Egypt

**DOI:** 10.1371/journal.pone.0069803

**Published:** 2013-07-29

**Authors:** Hiam Chemaitelly, Laith J. Abu-Raddad, F. DeWolfe Miller

**Affiliations:** 1 Infectious Disease Epidemiology Group, Weill Cornell Medical College - Qatar, Doha, Qatar; 2 Department of Public Health, Weill Cornell Medical College, New York, New York, United States of America; 3 Vaccine and Infectious Disease Division, Fred Hutchinson Cancer Research Center, Seattle, Washington, United States of America; 4 Department of Tropical Medicine and Medical Microbiology and Pharmacology, University of Hawaii, Honolulu, Hawaii, United States of America; University of North Carolina School of Medicine, United States of America

## Abstract

**Background:**

Egypt has by far the largest hepatitis C virus (HCV) prevalence in the world with 14.7% of the population being antibody positive for HCV. The aim of this study was to examine the association between knowledge of HCV and HCV antibody positivity among the Egyptian population.

**Methods:**

We characterized different measures of HCV knowledge and examined their associations with HCV prevalence, by analyzing a nationally representative database using standard epidemiologic methods. The database, the 2008 Egyptian Demographic and Health Survey, included demographic, health, and HCV biomarker information for a sample of over 12,000 individuals.

**Results:**

Basic knowledge of HCV was found to be high, but multiple gaps were identified in the specific knowledge of HCV and its modes of transmission. There was no statistically significant difference in HCV prevalence between those who have heard of HCV infection and those who have not (14.4% vs. 15.9%, p>.05). Similar results were found for the other HCV knowledge measures including those specific to HCV modes of transmission and to the sources of information for HCV awareness. Logistic regression analyses did not demonstrate an association between HCV knowledge and HCV prevalence.

**Conclusions:**

Our results do not provide support for an effect of awareness on reducing the risk of HCV infection in Egypt. Public health messages directed at the lay public may not provide sufficient empowerment for individuals to avoid HCV infection, and should be complemented with prevention programs to promote and strengthen infection control in the settings of exposure, particularly in health care facilities.

## Background

Hepatitis C virus (HCV) is a global public health challenge [1]. HCV is a blood-borne RNA viral infection that is mainly transmitted through direct exposure to infected blood such as through sharing of injections, blood transfusions, and accidental percutaneous exposures common in certain health-care professions [1], [2]. Prevention of HCV transmission is predicated on well-established infection control procedures for the prevention of blood-borne pathogens in health care delivery systems throughout the world [1], [2]. These procedures include the screening of blood supplies for HCV, in addition to other measures designed for specific health-care settings such as hemo-dialysis centers where reports of HCV outbreaks have been documented [3]. There are also well-developed HCV prevention interventions among population groups at high-risk of acquiring the infection such as injecting drug users [4]. On the other hand, while HCV transmission from mother to newborn is not frequent, interventions to reduce such transmission have yet to be clearly identified [5].

Egypt has by far the largest national-level HCV prevalence in the world [6], [7]. The estimated percentage of the Egyptian population in the 15–59 years age group who are positive for HCV antibody is 14.7% [8]. Over 80% of HCV infections in the Egyptian population are among individuals aged 30 years and above [8]. The re-use of needles during the parenteral antischistosomal therapy campaigns in the 1960s and 1970s is a major driver of the scale of this epidemic in Egypt [9]. Current HCV incidence appears to be driven by exposures within the health care system [8], [10]–[14]. The scale of the HCV problem in Egypt suggests the need for a specific epidemiologic strategy to prevent HCV transmission at a national level.

One public health measure in Egypt has been raising awareness over the last decade about HCV by both governmental and non-governmental stakeholders [15], [16]. As of 2005, 80.6% of women responding to the Egyptian Demographic and Health Survey (EDHS) had heard about “hepatitis C illness” [17], probably one of the highest levels globally. Nevertheless, widespread social engagement, such as seen in other countries regarding the HIV/AIDS epidemic, has not yet emerged in Egypt despite the severity of the epidemic [18].

A search of the literature to date has not found any published investigations on the evaluation of HCV awareness efforts in Egypt as a prevention program. Although such an assessment can only be implemented using a prospective study design, some insights about the link between HCV awareness and risk of HCV infection can be gained by analyzing cross-sectional data. To this end, we characterized in this work HCV-related knowledge among the Egyptian population and examined the association between HCV awareness and HCV antibody prevalence. The overarching aim of this study is to examine whether there is any signature that can support whether a higher awareness of HCV infection in Egypt could have contributed to a lower risk of HCV infection.

## Materials & Methods

The EDHS 2008 national survey collected demographic and health information from a nationally representative sample of the resident Egyptian population with a large sample size and using a rigorous sampling methodology [8]. Details related to the EDHS sampling design, sample size, study instruments, data collection, how informed consent was obtained, and other related methodology can be found in *El-Zanaty et al*. [8]. The EDHS data were downloaded with permission from Measure DHS [19]. Unique to this survey is the inclusion of HCV testing: out of 12,008 participants invited to give a blood specimen, 11,523 were tested for HCV. Positive third-generation ELISA results were retested, and all dual-positives were confirmed by chemiluminescent microplate immunoassay. Quantitative real time RT-PCR was used at the Egyptian Ministry of Health and Population Central Laboratory for the detection of HCV RNA. A stringent quality control system was also carried out in a separate government laboratory. Further methodological details related to specimen handling and laboratory methods employed for the detection of HCV antibody and HCV RNA are also described in *El-Zanaty et al.* [8]. The methodology used for the EDHS HCV serology testing is robust, and it is unlikely that another cross-reacting Flavivirus circulating in Egypt would have affected the sensitivity or specificity of the HCV ELISA assay [6].

For the purpose of this study, we merged the EDHS individual database with the HCV biomarker database based on established guidelines for managing DHS data [20]. All individuals with a test result for HCV antibody were included in our analysis. We specifically characterized information related to exposure to HCV knowledge that was collected in the EDHS. A list of the relevant HCV knowledge questions along with the number label provided for each question (Q) are presented in Table S1 of the Supporting Information.

We subsequently examined the association between exposure to HCV knowledge and HCV antibody positivity using prevalence odds ratios (ORs) and their respective 95% confidence intervals (CIs). These were derived from the implementation of chi square tests and bivariate logistic regression analyses (Table 1). P-values less than 0.05 were considered statistically significant.

**Table 1:** Prevalence of HCV antibody by response to HCV knowledge-related questions.

Variable	Distribution	HCV positive	Odds ratio	95% Confidence interval	p-value
	(%) N = 11,126	(%)			
*Question 601 “Have you ever heard of hepatitis C illness?”*					
Yes	82.1	14.4	1.00		
No	17.9	15.9	1.12	0.97–1.31	0.126
*Question 601 “Have you ever heard of hepatitis C illness?”*					
**Among urban residents**					
Yes	86.4	10.3	1.00		
No	13.6	10.6	1.04	0.74–1.47	0.822
**Among rural residents**					
Yes	79.1	17.8	1.00		
No	20.9	18.6	1.05	0.89–1.24	0.554
*Question 603 “Where did you hear or see that information?”*					
Television only	55.8	13.6	1.00		0.063
Television and other	33.9	16.0	1.20	1.01–1.43	
Other than television	10.3	16.5	1.25	0.96–1.62	
*Question 604 “How is hepatitis C spread from one person to another?”*					
**Heterosexual sex**					
Yes	12.6	13.3	1.00		
No/Don’t know	87.4	14.5	1.11	0.90–1.37	0.336
**Homosexual sex**					
Yes	2.1	12.5	1.00		
No/Don’t know	97.9	14.4	1.17	0.73–1.89	0.510
**Blood transfusion**					
Yes	60.6	13.8	1.00		
No/Don’t know	39.4	15.2	1.12	0.98–1.28	0.088
**Unclean needles**					
Yes	50.9	13.6	1.00		
No/Don’t know	49.1	15.2	1.09*	0.97–1.23	0.164
**Sharp objects (e.g. razors)**					
Yes	34.9	14.5	1.00		
No/Don’t know	65.1	14.3	0.99	0.86–1.13	0.875
**Casual physical contact**					
Yes	14.2	15.3	1.00		
No/Don’t know	85.8	14.2	0.92	0.77–1.10	0.365
**Mother-to-child transmission**					
Yes	6.2	13.4	1.00		
No/Don’t know	93.8	14.4	1.09	0.83–1.44	0.539
**Other modes of transmission**					
Yes	0.4	19.0	1.00		
No/Don’t know	99.6	14.4	0.71	0.32–1.61	0.419
*Question 508 “The last time you had an injection, was the injection from a new, unopened package?”*					
Among those who reported the safety procedure at the time they had their last injection *(n = 1,417; 12.7%)*					
Yes	85.0	13.8	1.00		
No	15.0	12.5	0.89	0.55–1.43	0.630
*Question 509 “In the last 6 months have you heard, seen, or received any information about what people should do to be sure that injections are given safely?”*					
Yes	22.8	12.6	1.00		
No	77.2	15.2	1.24	1.07–1.43	0.004

* Un-weighted regression analysis due to failure to obtain a maximum likelihood using weighted data.

An age-stratified analysis examining the association of HCV knowledge variables with HCV antibody positivity among individuals aged 30 years and younger and those aged more than 30 years was also conducted (Table 2). The possibility of having age as an effect modifier for the association between knowledge of HCV modes of transmission and HCV positivity was also explored. To this end, we performed a likelihood ratio test that compares the results of the logistic regression model that includes age and each one of the knowledge of HCV modes of transmission variables with an interaction term, to the parsimonious model without the interaction term.

**Table 2:** Age-stratified analysis for the association between HCV knowledge-related questions and HCV antibody positivity among the Egyptian population.

Variable	Distribution (%) N = 11,126		HCV positive (%)			Odds ratio (95% Confidence interval)	
*Question 601 “Have you ever heard of hepatitis C illness?”*							
**Adults < = 30 years**							
Yes	81.1		5.0			1.00	
No	19.0		6.4			1.29 (0.95–1.76)	
**Adults >30 years**							
Yes	83.3		23.8			1.00	
No	16.7		27.0			1.18 (0.98–1.42)	
*Question 604 “How is hepatitis C spread from one person to another?”*							
	Adults < = 30 years	Adults >30 years	Adults < = 30 years	Adults >30 years		Adults < = 30 years	Adults >30 years
**Heterosexual sex**							
Yes	11.8	13.3	4.1	21.5		1.00	1.00
No/Don’t know	88.1	86.7	5.2	24.1		1.27 (0.78–2.06)	1.16 (0.91–1.48)
**Homosexual sex**							
Yes	1.6	2.6	9.1	14.7		1.00	1.00
No/Don’t know	98.4	97.4	5.0	24.0		0.52 (0.21–1.33)	1.83 (1.04–3.22)
**Blood transfusion**							
Yes	60.2	61.1	4.8	22.8		1.00	1.00
No/Don’t know	39.8	38.9	5.5	25.3		1.17 (0.87–1.56)	1.15 (0.98–1.34)
**Unclean needles**							
Yes	51.5	50.2	4.8	22.8		1.00	1.00
No/Don’t know	48.5	50.0	5.4	24.7		1.14 (0.86–1.52)	1.11 (0.96–1.30)
**Sharp objects (e.g. razors)**							
Yes	34.4	35.3	4.6	24.2		1.00	1.00
No/Don’t know	65.6	64.7	5.3	23.5		1.17 (0.86–1.60)	0.96 (0.82–1.13)
**Casual physical contact**							
Yes	14.1	14.3	5.8	24.7		1.00	1.00
No/Don’t know	85.9	85.8	4.9	23.6		0.84 (0.57–1.24)	0.94 (0.76–1.16)
**Mother-to-child transmission**							
Yes	6.5	6.0	7.3	20.1		1.00	1.00
No/Don’t know	93.5	94.0	4.9	24.0		0.66 (0.39–1.09)	1.26 (0.89–1.77)
**Other modes of transmission**							
Yes	0.3	0.5	0	31.9		NA*	1.00
No/Don’t know	99.7	99.5	5.06	23.7		NA*	0.66 (0.27–1.65)
*Question 509 “In the last 6 months have you heard, seen, or received any information about what people should do to be sure that injections are given safely?”*							
Yes	23.3	22.2	4.3		21.8	1.00	1.00
No	76.7	77.8	5.6		25.0	1.32 (0.96–1.83)	1.20 (1.01–1.42)

* NA: Not Applicable.

We further performed an age-stratified multivariate logistic regression analysis to quantify the association between basic HCV knowledge and HCV antibody positivity among adults aged 30 years and younger and those aged more than 30 years (Table 3). The multivariate analysis was adjusted for sex (male/female), educational attainment (no education/primary/secondary/higher education), and place of residence (rural/urban).

**Table 3:** Age-stratified multivariate logistic regression for the association between HCV antibody prevalence as dependent variable and question 601 (Q601) “Have you ever heard of hepatitis C illness?” controlling for sex, educational attainment and place of residence.

Independent variables	Odds Ratio	95% Confidence interval	p-value
Among adults aged 30 years and below			
Q601 (no)	1.02	0.74–1.41	0.900
Sex (male)	1.56	1.20–2.03	0.001
Educational attainment (higher education)	1.00		
Secondary education	1.45	0.95–2.21	0.088
Primary education	2.30	1.32–4.02	0.003
No education	3.14	1.87–5.27	0.000
Place of residence (rural)	1.80	1.29–2.52	0.001
Among adults above 30 years of age			
Q601 (no)	0.99	0.81–1.21	0.950
Sex (male)	1.81	1.56–2.09	0.000
Educational attainment (higher education)	1.00		
Secondary education	1.17	0.90–1.52	0.246
Primary education	1.59	1.20–2.11	0.001
No education	1.87	1.42–2.46	0.000
Place of residence (rural)	1.92	1.63–2.26	0.000

We also constructed a composite score of the knowledge of HCV modes of transmission variables including blood transfusion, unclean needles, infected sharp objects, mother-to-child transmission, and lack of HCV transmission through casual physical contact. In each of these variables, correct answers were coded “1” while non-correct answers were coded “0”. We then conducted an age-stratified multivariate logistic regression to examine the impact of one-point increase in the knowledge score on HCV antibody positivity controlling for sex, educational attainment, and place of residence (Table 4).

**Table 4:** Age-stratified multivariate logistic regression for the association between HCV antibody prevalence as dependent variable and knowledge of HCV modes of transmission score (with higher score indicating a higher knowledge level) controlling for sex, educational attainment and place of residence.

Independent variables	Odds Ratio	95% Confidence interval	p-value
Among adults aged 30 years and below			
Knowledge of HCV modes of transmission score*	1.00	0.88–1.14	0.974
Sex (male)	1.68	1.25–2.26	0.001
Educational attainment (higher education)	1.00		
Secondary education	1.36	0.88–2.11	0.170
Primary education	2.12	1.14–3.94	0.018
No education	3.07	1.73–5.45	0.000
Place of residence (rural)	1.97	1.35–2.87	0.000
Among adults above 30 years of age			
Knowledge of HCV modes of transmission score*	1.01	0.94–1.08	0.816
Sex (male)	1.94	1.65–2.29	0.000
Educational attainment (higher education)	1.00		
Secondary education	1.22	0.93–1.60	0.143
Primary education	1.69	1.25–2.28	0.001
No education	1.89	1.41–2.53	0.000
Place of residence (rural)	1.87	1.57–2.24	0.000

* Knowledge of HCV modes transmission score includes knowledge of HCV modes transmission through blood transfusion, unclean needles, infected sharp objects, mother-to-child transmission, and lack of transmission through casual physical contact. Correct answers were coded “1” while non-correct answers were coded “0”.

We applied the sampling weights available in the EDHS databases in all of our statistical calculations. The data were analyzed using the statistical software of Epi Info 2000 and STATA/SE version 11 (Stata Corp., 2009, College Station, TX, USA).

## Results

The EDHS was based on a nationally representative sample of 16,527 women aged 15 to 49 and a further subsample of 6,578 women and 5,430 men aged 15 to 59 (n = 12,008), of which, 11,523 completed the HCV biomarker testing. General information related to the level of knowledge about HCV by socio-demographic attributes can be found in the EDHS report [8].

Table 1 presents different measures of HCV knowledge and the association between measures of HCV knowledge and HCV antibody positivity. The table shows that 82.1% (95% CI: 81.3–82.9%) of the sample responded that they had heard of HCV. However, there was no statistically significant difference in HCV prevalence between individuals who have heard of HCV and those who have not heard of HCV (14.4% vs. 15.9%, p-value of 0.126). Individuals who have not heard of HCV had 12% higher odds of being HCV infected compared to their counterparts (95% CI: 0.97–1.31%). Similarly, there was no statistical difference in HCV positivity by knowledge of HCV when stratified by place of residence, with a higher exposure to HCV knowledge among residents of urban areas versus residents of rural areas (86.4% vs. 79.1%). HCV positivity was higher among individuals reporting receiving HCV information from multiple sources, however, the association was either not significant or borderline significant with limited epidemiological relevance (Table 1).

A substantial proportion of the Egyptian population had a correct knowledge regarding the potential for HCV transmission through blood transfusions (60.6%) and sharing of unclean needles (50.9%). Still, the majority were not familiar with other routes of HCV transmission such as sharp objects (34.9%) and mother-to-child transmission (6.2%). Some also had misconceptions in relation to specific modes of HCV transmission such as the belief that HCV can spread through casual physical contact (14.2%). In addition to the variability in the knowledge of HCV modes of transmission, we found no significant differences in the levels of HCV knowledge among HCV infected individuals and those not infected with HCV (Table 1).

Only 12.7% of the total sample (n = 1,417) reported the safety procedures that were followed the last time they received an injection. Of these, 85% confirmed that the last injection they received was from a new unopened package. No statistically significant difference in HCV antibody positivity was observed among those who did and those who did not report proper safety procedures the last time they received an injection (13.8% vs. 12.5%, respectively, p-value of 0.630).

On the other hand, 22.8% of the total sample indicated that they had received information in the last six months on injection safety. Lower levels of HCV positivity were observed among this group compared to the larger group who were not exposed to injection safety information in the last six months (12.6% vs. 15.2%, respectively). The association was statistically significant (OR: 1.24, 95%: CI 1.07–1.43) at the bivariate level, but did not reach statistical significance (OR: 1.14, 95%: CI 0.98–1.32) in the multivariate analysis after controlling for sex, educational attainment, and place of residence (analysis not shown).

An age-stratified analysis revealed higher HCV antibody positivity among adults older than 30 years of age compared to younger adults (Table 2). Overall, minor or no differences were observed in the association between the different aspects of HCV knowledge and HCV positivity among the younger (≤30 years) versus the older adults (>30 years) (Table 2). Including age as an effect modifier for the association between knowledge of HCV modes of transmission variables and HCV antibody positivity did not demonstrate better explanatory power to understand the apparent lack of association between HCV knowledge and HCV infection.

Table 3 shows the results of an age-stratified multivariate logistic regression where exposure to basic HCV knowledge (Q601) is included in the model as a main independent variable along with potential confounders such as sex, educational attainment, and place of residence. The analysis revealed a lack of association between exposure to this HCV knowledge measure and HCV antibody positivity among both younger and older adults. Conversely, moderate to strong associations were observed between male-sex, lower educational attainment, and being a resident of a rural area and HCV antibody positivity among both age groups (Table 3).

Another age-stratified multivariate logistic regression examining the association between a composite score of the knowledge of HCV modes of transmission variables as a main independent variable, and HCV positivity controlling for sex, educational attainment, and place of residence also showed that a point increase in the knowledge score was not associated with HCV antibody positivity among both younger and older adults (Table 4). The analysis also demonstrated that male-sex, lower educational attainment, and being a resident of a rural area are main risk factors for HCV infection among both age groups (Table 4).

## Discussion

We characterized different aspects of HCV knowledge among the population of Egypt; the country that has by far the highest HCV prevalence in the world. We also explored associations between different aspects of HCV knowledge and HCV infection. Our analyses indicated that generally basic knowledge of HCV is high in Egypt, not a surprising result considering the scale of the HCV epidemic in this country and its associated disease morbidity and mortality. Despite basic knowledge, comprehensive knowledge of HCV and its modes of transmission cannot be described as satisfactory in a country with such a level of disease burden. Our analyses also generally revealed no significant differences in HCV-related knowledge among HCV infected and HCV uninfected individuals in both the younger and older adult generations in Egypt. There was also no discernible signature for major differences in the relationship between the various aspects of HCV knowledge and HCV infection among the younger versus the older adults.

Media outlets constitute the main sources of HCV knowledge with television appearing to be the primary source. Exposure to media might also explain the differences in exposure to HCV knowledge among urban versus rural residents. These findings pose questions related to the quality and type of information (therapeutic vs. preventive) delivered to the average individual via media channels. There is need to engage the media as key actors in HCV prevention efforts, but through dissemination of accurate prevention information that can empower individuals to avoid HCV infection.

While the majority of the Egyptian population reported ever hearing of HCV, a substantial proportion had poor levels of knowledge in relation to several HCV modes of transmission. Though a considerable proportion of the population is aware that HCV can be transmitted through blood transfusions and sharing of unclean needles, many were not able to recognize sharp objects and mother-to-child transmission as potential modes of HCV transmission. Others had also misconceptions regarding HCV transmission. This highlights the need for both governmental and non-governmental stakeholders working on raising HCV awareness among the public to reinforce knowledge related to the modes of transmission.

The lack of association between the different measures of HCV knowledge and HCV infection do not, collectively, provide support for an effect of awareness on reducing the risk of HCV infection. Public health messages directed at the lay public do not appear to provide sufficient empowerment for individuals to avoid HCV infection. An alternative explanation for the lack of association is that most HCV infected persons were infected before they became aware of HCV, such as during the parenteral antischistosomal therapy campaigns in the 1960s and 1970s [9]. Another explanation is that new HCV infections are acquired within the context of the health care delivery systems [7], [8], [10]–[15], and hence, may be beyond the individual’s ability to control regardless of knowledge. Irrespective of the explanation, the lack of association indicates that awareness programs cannot be sufficient to prevent HCV transmission in Egypt, and must be complemented with robust prevention programs to promote and strengthen infection control in the settings of exposure, particularly in health care facilities.

The effectiveness of infection control procedures for the prevention of blood-borne pathogens in health care delivery systems is well established [1]–[3]. Substantial reductions in HCV incidence can be achieved by prioritizing prevention approaches that target specifically the actual settings at which infected individuals may pass the infection to uninfected individuals, that is mainly the health care system [15]. This is particularly true in Egypt in light of the documented need for improved injection safety and infection control [21], [22]. One success story to this end is the infection control programs implemented by the Egyptian Ministry of Health and Population in dialysis centers across Egypt [15]. The programs yielded a substantial decrease in HCV incidence from 28% to 6% among these patients [15].

Nevertheless, robust infection control programs that are affordable and cost-effective in resource-rich settings may not be affordable or cost-effective in a resource limited-setting such as Egypt. For instance, a national HCV prevention intervention program that costs only 10 US dollars per person per year would require the allocation of close to one billion dollars in a country of over 80 million inhabitants such as Egypt. This translates to almost half the annual budget of the Ministry of Health and Population in this country [7], which is already burdened by other needs and by the high cost of providing treatment to HCV infected individuals. The latter treatment program alone consumes about 20% of the Ministry of Health and Population total annual budget [15]. Rigorous cost-effectiveness analyses are needed to examine the impact and feasibility of different potential infection control programs.

The principal objective of this investigation was to explore the association between HCV knowledge and HCV antibody positivity among the Egyptian population, and to explore whether such association is consistent with an assumed effect for awareness on reducing the risk of HCV infection. Our findings did not provide support for such an effect, but our approach is pragmatic and empirical rather than etiologic. An etiologic investigation of public awareness and HCV transmission would require HCV incidence estimation in a representative sample ascertained to be free of HCV and followed for an extended period of time. Such an investigation is challenging by design and may not be justified ethically, especially in the context of a randomized clinical trial, given that the modes of HCV transmission are well established.

Our interpretation of the results of this analysis is limited by the cross-sectional design of the EDHS survey. For any given individual in the survey who was identified as HCV antibody positive, the times of exposure to HCV awareness and of acquisition of the HCV infection cannot be determined. Causal associations cannot be established. Our analysis is also limited by the structure of the HCV knowledge-related questions in the EDHS. Some of the questions are not sufficiently well-defined nor are tied to a specific context or a time frame. Nevertheless, our analyses are indicative of the need for more effective and targeted HCV awareness efforts, and more so, emphasis on infection control in the settings of exposure, particularly in health care facilities.

To validate our findings, we conducted multiple analyses using different HCV knowledge-related questions probing different aspects of HCV knowledge. All analyses consistently showed a lack of association between HCV knowledge and HCV prevalence among both younger and older adults. We also conducted sensitivity analyses to assess the robustness of our findings to potential biases. It is conceivable that any individual respondent could answer “yes” to Q601, “Have you ever heard of hepatitis C illness?” out of social desirability. Such bias would reduce the specificity of the question. To this end, we conducted a simulation where the specificity and sensitivity to this question was modified (not shown). Different simulations created changes in the percentage of the population that answered “yes”. Yet, the results revealed persistently a lack of association between exposure to HCV knowledge and HCV antibody positivity. This was expected as misclassification bias, artificial or real, regarding an individual’s response, had no effect on the specificity and sensitivity of the HCV antibody ELISA assays which had been completed independently, and at a later time after the interview.

### Conclusions

Our results did not demonstrate an association between HCV knowledge and HCV infection among the adult population of Egypt. This finding does not provide support for an effect of awareness on reducing the risk of HCV infection. Public health messages directed at the lay public may not provide sufficient empowerment for individuals to avoid HCV infection, and should be complemented with robust prevention programs to promote and strengthen infection control in the settings of exposure. Substantial reductions in HCV incidence among the Egyptian population can only be achieved through a combined approach that reinforces both targeted and effective HCV awareness and infection control in the health care delivery systems. Additional studies are needed to evaluate the impact and cost-effectiveness of potential prevention programs on reducing HCV transmission in this country.

## Acknowledgments

The authors are thankful for El-Zanaty and associates for conducting the 2008 Egypt Demographic and Health Survey study, under the endorsement of the Ministry of Health and Population in Egypt, and in collaboration with ICF International. The authors are also thankful for Measure DHS for putting these data in the service of science, and for the U.S. Agency for International Development and other donors for supporting these initiatives.

## Supporting Information

Table S1 **Knowledge of hepatitis C virus questions as collected in the Egyptian Demographic and Health Survey, 2008.**
(DOC)
