# Supplementary material for: An Apparent Lack of Epidemiologic Association between Hepatitis C Virus Knowledge and the Prevalence of Hepatitis C Infection in a National Survey in Egypt
Source: PLoS One. 2013 Jul 29;8(7):e69803. doi: 10.1371/journal.pone.0069803 (PMC3726777; doi:10.1371/journal.pone.0069803)
Supplement: Table S1 — Knowledge of hepatitis C virus questions as collected in the Egyptian Demographic and Health Survey, 2008. (DOC) [file pone.0069803.s001.doc]

**Supporting Information File**

**Table S1. Knowledge of hepatitis C virus questions as collected in the Egyptian Demographic and Health Survey, 2008** .

| *Question 508:* “The last time you had an injection from a health worker, did the person who gave you that injection take the syringe and needle from a new, unopened package?” |
| --- |
| *Question 509:* “In the last 6 months have you heard, seen, or received any information about what people should do to be sure that injections are given safely?” |
| *Question 601:* “Have you ever heard of hepatitis C illness?” |
| *Question 602:* “Have you heard information about hepatitis C in the last six months?” |
| *Question 603:* “Where did you hear or see that information?” |
| *Question 604:* “How is hepatitis C spread from one person to another?” |
